# Soy Isoflavones Regulate Lipid Metabolism through an AKT/mTORC1 Pathway in Diet-Induced Obesity (DIO) Male Rats

**DOI:** 10.3390/molecules21050586

**Published:** 2016-05-03

**Authors:** Chao Huang, Dejiang Pang, Qihui Luo, Xiaolin Chen, Qi Gao, Liangqin Shi, Wentao Liu, Yuanfeng Zou, Lixia Li, Zhengli Chen

**Affiliations:** 1 Laboratory of Experimental Animal Disease Model, College of Veterinary Medicine, Sichuan Agricultural University, Chengdu 611130, China; huangchao@sicau.edu.cn (C.H.); lqhbiology@163.com (Q.L.); chenxl3984@163.com (X.C.); gaoqi12@126.com (Q.G.); shiliangqin87@163.com (L.S.); liuwt1986@126.com (W.L.); 2 Key Laboratory of Animal Disease and Human Health of Sichuan Province, College of Veterinary Medicine, Sichuan Agricultural University, Chengdu 611130, China; 3 Department of Biochemistry and Molecular Biology, West China School of Preclinical and Forensic Medicine, Sichuan University, Chengdu 610041, China; pangdejiang@gmail.com; 4 Natural Medicine Research Center, College of Veterinary Medicine, Sichuan Agricultural University, Chengdu 611130, China; yuanfengzou@sicau.edu.cn (Y.Z.); lilixia905@163.com (L.L.)

**Keywords:** soy isoflavones, obesity, lipid metabolism, mTORC1

## Abstract

The pandemic tendency of obesity and its strong association with serious co-morbidities have elicited interest in the underlying mechanisms of these pathologies. Lipid homeostasis, closely involved in obesity, has been reported to be regulated by multiple pathways. mTORC1 is emerging as a critical regulator of lipid metabolism. Here, we describe that the consumption of soy isoflavones, with a structural similarity to that of estradiol, could mitigate obesity through an AKT/mTORC1 pathway. Fed with soy isoflavones, the diet-induced obesity (DIO) male rats exhibited decreased body weight, accompanied with suppressed lipogenesis and adipogenesis, as well as enhanced lipolysis and β‑oxidation. The phosphorylation of AKT and S6 were decreased after soy isoflavone treatment *in vivo* and *in vitro*, suggesting an inhibition effect of soy isoflavones on mTORC1 activity. Our study reveals a potential mechanism of soy isoflavones regulating lipid homeostasis, which will be important for obesity treatment.

## 1. Introduction

Substantial literature has reported that overweight and obesity are major causes of co-morbidities [1,2], which can lead to further morbidity and mortality caused by diabetes [3,4], fatty liver [5], hypertension [6], cardiovascular diseases [7], and cancer, *etc.* [8]. Increasing clinical studies have detected damage, inflammation, and metabolic disorders in the liver of obesity patient, but the pathogenesis of obesity remains largely unknown [9,10]. The liver acts as a major organ for fatty acid metabolism, including synthesis, transport, and oxidation processes. The imbalance of these processes may cause excessive fatty acid accumulation, resulting in obesity.

mTOR is an evolutionarily-conserved serine/threonine kinase that exists within two functionally distinct protein complexes, the mechanistic target of rapamycin complexes 1 (mTORC1) and 2 (mTORC2) [11]. mTORC1 could sense systemic and local nutrient and energy availability, to control a myriad of cellular processes, such as protein synthesis and autophagy [12]. Moreover, mTORC1 is emerging with growing genetic and pharmacological evidence as a central regulator of lipid homeostasis, including lipid synthesis, oxidation, transport, storage, and lipolysis, as well as adipocyte differentiation and function [13]. The activation of mTORC1 is reported to enhance lipogenesis and adipogenesis, resulting in lipid storage [14,15]. Additionally, mice lacking mTORC1 activity in their livers, through genetic knockout or specific inhibitors, exhibit lipogenesis defects and enhanced lipolysis and β‑oxidation [16,17,18]. PPARs and SREBPs, known lipid metabolism regulators, are involved in the role of mTORC1 in lipid homeostasis [14,19,20]. However, the exact mechanisms in these processes are not yet well defined.

Soy has been a regular part of the diet in many countries for centuries, and the consumption of soy has been generally considered beneficial [21]. Soy isoflavones, the naturally phytoestrogen components of soy, are thought to be responsible for the beneficial effects of soy, with a potentially protective effect against a number of diseases, such as breast cancer, osteoporosis, and hypercholesterolemia [22]. Recently, researchers have found that soy isoflavones could influence the lipid metabolism due to their structural similarity to that of estradiol. However, the effects of soy isoflavones on these processes in both humans and animals, especially in males, are not fully understood. Here, we show that soy isoflavones could mitigate obesity through an AKT/mTORC1 pathway in diet induced obesity (DIO) male rats. The DIO rats, supplemented with soy isoflavones, have shown a decreased body weight and less accumulation of lipids in livers, which result from a suppressed lipogenesis and adipogenesis, as well as an enhanced lipolysis and β‑oxidation. The phosphorylation of AKT and S6 are suppressed after soy isoflavones treatment, suggesting an inhibition effect of soy isoflavones on mTORC1 activity. Genistein and daidzein, common components of soy isoflavones, attenuate the activation of mTOCR1, and reduce lipid accumulation induced by oleic acid in HepG2 cells. These results reveal a new perspective for soy isoflavones in obesity treatment.

## 2. Results

### 2.1. Soy Isoflavones Exhibited Remarkable Effects on Body Weight and Adiposity in DIO Male Rats

**Figure 1:**
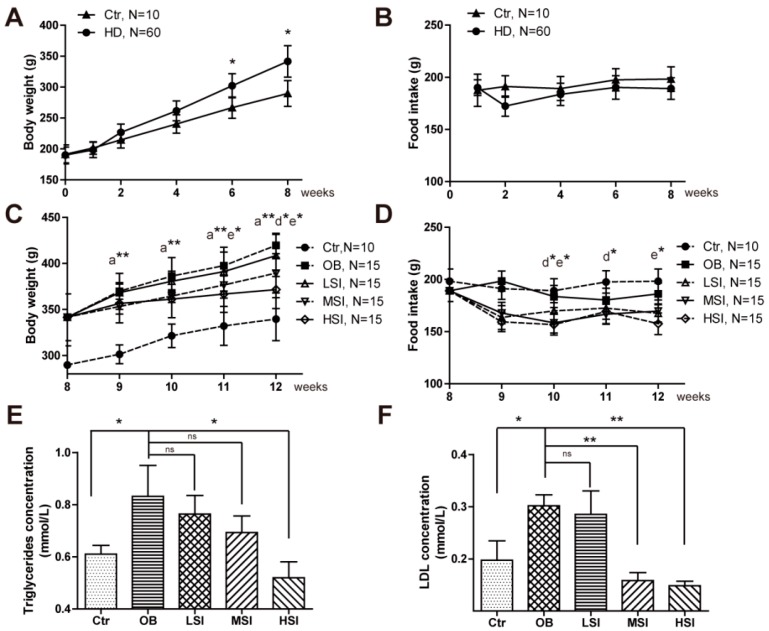
Soy isoflavones reduce the body weight, as well as the plasma TG and LDL concentrations. (**A**) Quantification shows the body weight of rats feed with basal diets and high fat diets; (**B**) Quantification shows no difference in food intake of rats feed with basal diets and high fat diets; (**C**) Quantification shows the body weight trend of DIO rats feed with basal diets and the addition with different doses of soy isoflavones. a**: b *vs.* a, *p* < 0.01; d*: b *vs.* d, *p* < 0.05; e*: b *vs.* e, *p* < 0.05; (**D**) Quantification shows the food intake trend of DIO rats feed with basal diets and the addition with different doses of soy isoflavones. d*: a *vs.* d, *p* < 0.05; e*: a *vs.* e, *p* < 0.05; (**E**) Quantification shows the triglycerides concentration of DIO rats feed with basal diets and the addition with different doses of soy isoflavones; (**F**) Quantification shows the LDL concentration of DIO rats feed with basal diets and the addition with different doses of soy isoflavones. Error bars indicate SEM. Significant difference in: ^a^ control group, ^b^ obesity group, ^c^ low-dose soy isoflavones group, ^d^ middle-dose soy isoflavones group, ^e^ high-dose soy isoflavones group. ns, no statistical significance, * *p* < 0.05, ** *p* < 0.01.

To examine the biological functions of soy isoflavones in male, we generated the DIO male rats with high fat diets. After seven-week feeding, they gained more body weight than the control ones (Figure 1A), and there was no statistical difference of food intake between the control and the DIO groups (Figure 1B). Then, the DIO male rats were randomly divided into the obesity control group (OB, hereafter) fed with the normal diet and the soy isoflavonesgroups fed with both the normal diet and different doses of soy isoflavones. We found that the middle dose of soy isoflavones (MSI, hereafter) and the high dose of soy isoflavones (HIS, hereafter) could significantly reduce the body weight of DIO male rats (Figure 1C), and there was no statistical difference of body weight between the low dose of soy isoflavones group (LSI, hereafter) and the OB group (Figure 1C), which was not consistent with the previous work done with Obese Male Zucker Diabetic Fatty (ZDF) rats [23]. Food intake was slightly reduced in MSI and HSI groups compared with that of the control group, and no statistical difference was found between the OB group and the soy isoflavones groups (Figure 1D). Obesity is always accompanied with high levels of triglycerides (TG, hereafter) and low-density lipoprotein (LDL, hereafter) in plasma. We observed a remarkable reduction of TG and LDL concentrations in the soy isoflavones groups, especially in MSI and HIS ones (Figure 1E,F). These data indicated that soy isoflavones could mitigate obesity in male rats.

### 2.2. Soy Isoflavones Could Reduce Lipid Accumulation in Livers of DIO Rats

**Figure 2:**
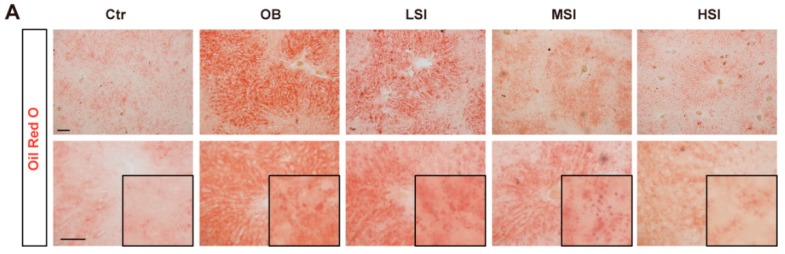

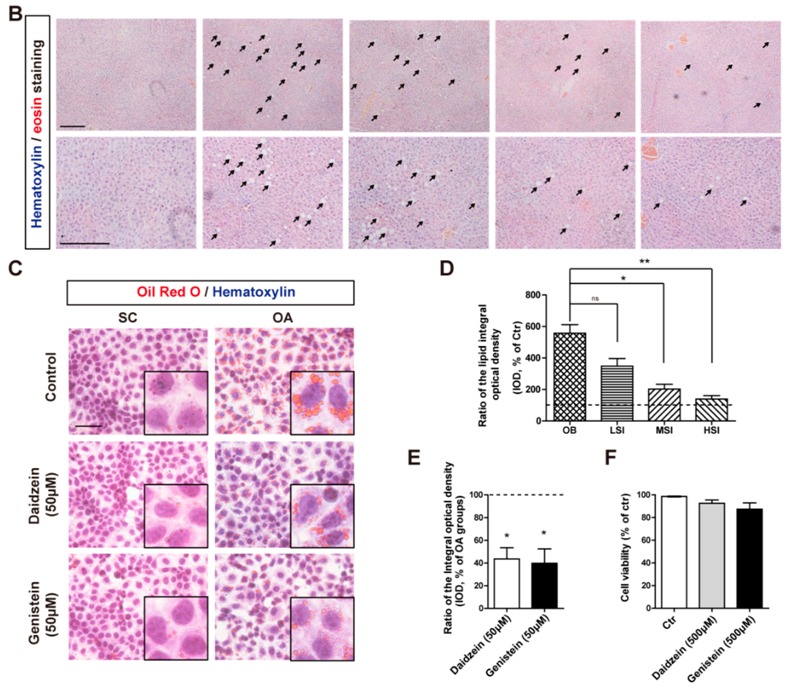
Soy isoflavones suppress the lipid accumulation *in vivo* and *in vitro*. (**A**) Representative images of Oil Red O staining show the accumulation lipid in the liver of rats feed with different diets. The insets show enlarged lipid droplets. Bar: 50 μm; (**B**) Representative images of H and E staining show the steatosis (arrow indicated) in the liver of rats feed with different diets. Bar: 100 μm; (**C**) Representative images of Oil Red O staining show the accumulation lipid, induce by 1 mM OA for 24 h, following a 24 h daidzein and genistein treatment in HepG2 cells. SC, solvent control; OA, oleic acid. The insets show enlarged lipid droplets. Bar: 50 μm; (**D**) Quantification shows a significant decrease in the ratio of the lipid integral optical density between OB groups and soy isoflavone-addition groups. The dashed shows the ctr; (**E**) Quantification shows a significant decrease in the ratio of the lipid integral optical density in daidzein- and genistein-treated HepG2 cells. * *p* < 0.05, ** *p* < 0.01; (**F**) Quantification shows the cell viability of HepG2 cells treated with daidzein and genistein. Error bars indicate SEM. *n* = 3.

Acting as a major organ for fatty acid metabolism, the liver is always abnormal in obesity patients. Fed with the high fat diet, the rats have shown an increased accumulation of lipids in livers with Oil Red O staining (Figure 2A,D), while the addition of soy isoflavones to the diet could significantly improve this phenotype in a dose-dependent manner (Figure 2A,D). Hematoxylin and eosin staining has also shown an increase of steatosis in the OC group, and less steatosis cells were detected in the soy isoflavones-fed groups (Figure 2B). Furthermore, daidzein and genistein, the common components of soy isoflavones, could obviously reduce the lipid accumulation induced by oleic acid (OA) in HepG2 cells (Figure 2C,E), and they did not affect the viability of cells (Figure 2F). These results suggested that soy isoflavones are involved in the lipid metabolism of hepatocyte cells.

### 2.3. Effects of Soy Isoflavones on the Lipid Metabolism in the Liver and Adipose Tissue

**Figure 3:**
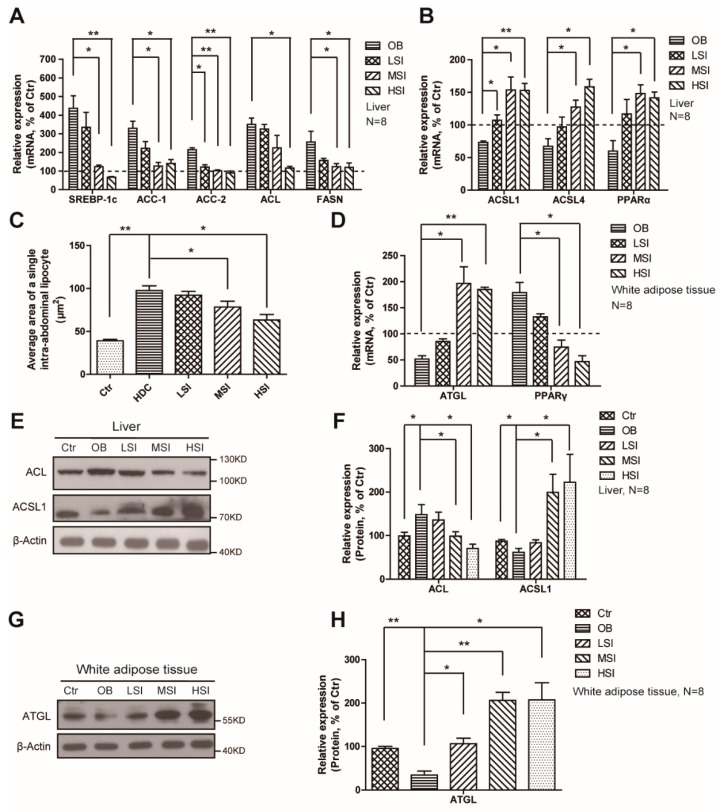
Soy isoflavones suppress lipogenesis and adipogenesis, and enhance lipolysis and β‑oxidation in DIO rats. (**A**) qRT-PCR shows decreased mRNA levels of the lipogenesis-related genes as indicated in liver of the soy isoflavone-fed DIO rats in comparison to the OB group; (**B**) qRT-PCR shows increased mRNA levels of the β‑oxidation related genes as indicated in liver of the soy isoflavone-fed DIO rats in comparison to OB group; (**C**) Quantification shows changes of the average area of a single intra-abdominal lipocyte in rats fed different diets; (**D**) qRT-PCR shows increased mRNA levels of ATGL, while a decreased mRNA levels of PPARγ, as indicated in white adipose tissue of the soy isoflavone-fed DIO rats in comparison to the OB groups; (**E**,**F**) Western blots and quantification show an increase in ACL, and a decrease in ACSL1 in the liver of the soy isoflavone- fed DIO rats in comparison to the OB group; (**G**,**H**) Western blots and quantification show an increase in ATGL in the liver of the soy isoflavone-fed DIO rats in comparison to the OB group. Error bars indicate SEM. * *p* < 0.05, ** *p* < 0.01. The dashed shows the ctr.

Lipid metabolism refers to the processes that involve the synthesis and degradation of lipids. Liver is the major site for fatty acid synthesis and β-oxidation, and the white adipose tissue is the place for lipid storage. We have found that the expression of SREBP1, the key transcript factor for lipogenesis, was upregulated in the liver after high-fat diet feeding, and so did its target genes of lipogenic enzymes, ACC-1, ACC-2, ACL, and FASN (Figure 3A). Moreover, dose-dependent soy isoflavones have suppressed expressions of these genes (Figure 3A). Meanwhile, the expressions of β-oxidation- and ketogenesis-related genes ACSL1, ACSL4, and PPAR-α were decreased in the OC group, while the soy isoflavones increased these expressions in livers (Figure 3B). In white adipose tissue, the high-fat diet inhibited the expressions of lipolysis related genes, ATGL (Figure 3D), resulting in an increase of the average area of a single lipocyte (Figure 3C). After treating with soy isoflavones, the rats exhibited increased expressions of ATGL (Figure 3D), and a decrease of the lipocyte’s average area (Figure 3C). PPARγ, the master regulator of terminal adipocyte differentiation, was upregulated after high-fat diet treatment, and exhibited a suppressed expression after soy isoflavones consumption (Figure 3D). Consistent with the gene expression, Western blots have revealed the same findings that the high fat diet treatment increased the protein level of ACL in liver, and reduced the protein levels of ACSL1 in liver and ATGL in white adipose tissue (Figure 3E–H). Meanwhile, the addition of soy isoflavones suppressed the protein level of ACL in the liver, and increased the protein levels of ACSL1 in the liver and ATGL in white adipose tissue (Figure 3E–H). These findings have revealed an important role of soy isoflavones in controlling lipid metabolism in many settings, both lipogenesis and lipolysis, in the male obese rats. However, more work needs to be done to reveal the mechanism for how soy isoflavones regulate these processes.

### 2.4. Soy Isoflavones Suppressed the Activity of mTORC1

**Figure 4:**
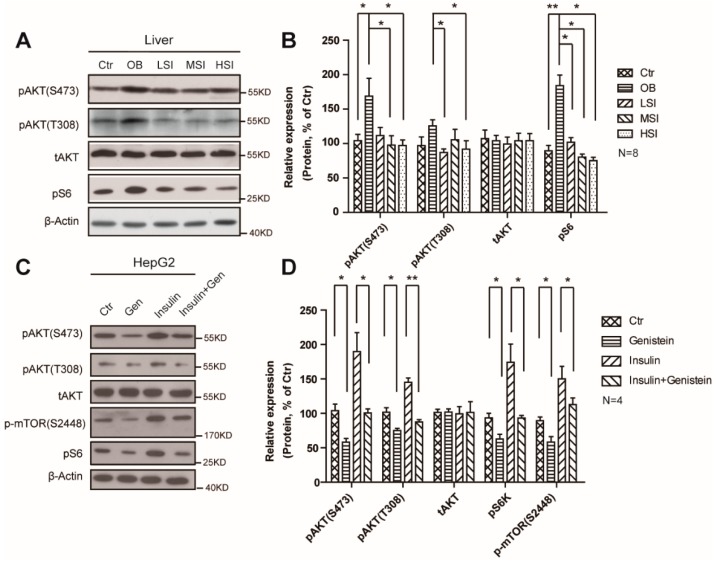
Soy isoflavones suppress the mTORC1 activity via AKT pathway. (**A**,**B**) Western blots and quantification show a suppression function of soy isolflavones on mTORC1 activity in DIO rats; (**C**,**D**) Western blots and quantification show a suppression function of soy isoflavones on mTORC1 activity in HepG2 cells. Error bars indicate SEM. * *p*< 0.05, ** *p* < 0.01.

Growing genetic and pharmacological evidence has demonstrated the functional importance of mTORC1 signaling in controlling mammalian lipid metabolism. Here, we showed that high-fat diet treatment increased mTORC1 activity in male rats, indicated by the phosphor-S6 protein levels (Figure 4A,B), and this induction occurs concurrently with the phosphorylation of AKT (S473 and T308) (Figure 4A,B). The addition of soy isoflavones could remarkably suppress the protein levels of phosphor-S6 and phosphor-AKT (S473 and T308), which represents a decrease in activity of mTORC1. Furthermore, we observed a suppression of phosphor-S6, phosphor-AKT, and phosphor–mTOR in genistein treated HepG2 cells (Figure 4C,D). Meanwhile, the phosphorylation of AKT and S6 was strongly increased in the insulin treated cells but not in the insulin and genistein treated ones (Figure 4C,D). These results display the inhibition function of soy isoflavones on mTORC1 activity.

## 3. Discussion

The present study shows that soy isoflavone-based diets mitigate the abnormalities in the high-fat diet induced obesity male rats. There are many disparities in the preview literatures account for the function of soy isoflavones in controlling body weight. Chen *et al.* [24] reported the positive regulation of isoflavones on body weight. We found the addition of soy isoflavones reduce the body the weight of DIO rats, accompanied with a decrease in plasma TG and LDL concentrations. However, these are inconsistent with some previous works in male pigs and ZDF rats, which have shown that the animals fed with soy isoflavones gain more body weight [23,24]. Moreover, the consumption of soy isoflavones has been implicated in the changes in many endocrinologic, physiologic, and metabolic processes that affect human health [25]. A meta-analysis has indicated that soy isoflavones reduce the LDL concentration, but not triacylglycerol, in hypercholesterolemic patients [26]. Our work has shown that the DIO male rats fed with soy isoflavones have lower plasma LDL and triacylglycerol levels. These discrepancies display the complicated function of soy isoflavones, or even estrogens, through different mechanism in mammalian metabolism.

Liver plays a major role in mammalian metabolism, especially in lipogenesis, lipid β-oxidation, and ketogenesis [27,28]. In this present study, the DIO rats fed with soy isoflavones have less lipid accumulation and steatosis in livers, which indicates that soy isoflavones may promote the clearance of lipid droplets in obese male rats. These results are consistent with previous studies showing that soy isoflavones could reduce the formation of hepatic lipid droplets in the female rats [29]. Murosaki *et al.* reported that soy isoflavone could reduce the lipid accumulation in 3T3-L1 cells after differentiation to adipocytes [30], and we have shown that the genistein and daidzein, the common components of soy isoflavones, could remarkably reduce the lipid accumulation induced by OA in HepG2 cells. These results suggest that soy isoflavones could be an effective way for controlling lipid metabolism.

Multiple research has revealed the potential mechanisms of soy isoflavones in regulating lipid metabolism. Most of them are focused on the transcription factor SREBPs, regulating metabolic enzymes, and the peroxisome proliferator-activated receptors (PPARs). We have found that the expression of SREBP1 and its downstream target genes (ACC, ACL, and FASN) are suppressed by soy isoflavone feed diets in DIO rats, which is consistent with previous works [31]. *In vitro* and *in vivo* studies have shown that soy isoflavones enhance the expressions and activity of PPARα and PPARγ [32,33,34,35], but we have found the DIO rats fed with soy isoflavones show increased PPARα expression in the liver and decreased PPARγ expression in white adipose tissue, accompanied with enhanced lipolysis and β-oxidation. These suggest that the soy isoflavones modulate the lipid metabolism via complicated mechanisms, which are PPAR-dependent and -independent. Genetic mouse models have also demonstrated that PPARs are sufficient, but not necessary, for soy isoflavones to regulate lipid metabolism [36]. Therefore, more work needs to be done to display the mechanisms that soy isoflavones are involved in.

It has been appreciated for some time that AKT/mTORC1 pathway plays a role in lipid metabolism, which regulates not only lipogenesis and lipolysis, but also adipogenesis and lipid transport [13]. Insulin activates mTORC1 through a pathway involving the Akt-mediated phosphorylation and inhibition of TSC2, which stimulates the global expressions of SREBP1 and SREBP2 targets and drives lipogenesis [37,38]. Moreover, mTORC1 seems to suppress ketogenesis and fatty acid β-oxidation via its regulation of N-CoR1 and PPARα [27,39], and promote pre-adipocytes to mature adipocytes through both its inhibition of 4E-BP and its activation of PPARγ via a poorly understood mechanism [15,40,41]. Here we present that soy isoflavones suppress the activity of mTORC1, with a reducing phosphorylation of AKT, in DIO rats. The inhibitory effects on mTORC1 of soy isoflavones may possibly be attributed to their competitive binding to the growth factor receptors, because soy isoflavones could remarkably attenuate the activation of mTORC1 and the phosphorylation of AKT induced by insulin. Similar to estrogens, dietary soy significantly reduced cerebral edema and vascular apoptosis 24 h after stroke, accompanied by decreased VEGF receptor activation and suppressed phosphorylation of AKT [42]. These results together suggest that the suppression function of soy isoflavones on AKT/mTORC1 activation is widespread, but not tissue-specific.

## 4. Materials and Methods

### 4.1. Animal Care and Maintenance

All animal work was done in accordance with the Animal Care and Use Committee Guidelines of Sichuan Agricultural University, China. Eighty male Sprague Dawley (SD) rats (five weeks old) (Dashuo, China) were maintained in individual cages in a specific pathogen-free environment with an automatically-controlled 12-h light/dark cycle and free access to food and water for seven days. Then, they were randomly divided into the basal diet group (Table 1) (Dashuo, Chengdu, China; control, Ctr, *n* = 10) and the high fat diet group (basal diet, 69.5%; pork fat, 15%, sucrose, 15%; pig bile, 0.5%; *n* = 70). These rats were treated with the indicated diets for eight weeks to induce obesity, and the body weight was measured weekly. The criterion for the DIO rats is that the body weight of the rats of the high-fat diet group is 1.4 times more than that of the control group. The DIO rats were further randomly divided into four groups (*n* = 15/group), and they were fed with the basal diet with different doses of soy isoflavone additions (soy isoflavone extracts, North China Pharmaceutical Co., Ltd., Shijiazhuang, China) as described in Table 2. The compounds of soy isoflavone extracts, as quantified by HPLC, are shown in Table 3. After four weeks of feeding, these rats were decapitated for the subsequent experiments.

### 4.2. Body Weight, Food Intake, and Plasma Measurements

Body weight and food intake were measured weekly. A blood sample was collected from the lateral tail vein. A 1- to 2-mm section was cut in the tip of the tail with a sterile scalpel blade. Blood was then milked from the base of the tail to the tip until a sufficient volume of blood was collected for blood biochemical analysis weekly (Beckman CX4, Indianapolis, IN, USA).

### 4.3. Histopathologic Evaluation

Part of the liver tissue was fixed in 4% paraformaldehyde before paraffin sections were performed. Then, the hematoxylin and eosin (H and E) staining was performed according to the instruction provided by the Manufacturer (Beyotime, Shanghai, China).

To detect the average area of a single lipocyte, adipose tissue frozen sections (30 µm) were prepared. The Sudan III staining was performed according to the instructions provided by the manufacturer (Beyotime). The average area of a single lipocyte was quantified by Image J software.

To detect lipid accumulation in the liver and HepG2 cells, liver frozen sections and HepG2 cells grow on cover glass were rinsed with distilled water, stained with 0.18% Oil-Red O (Sigma-Aldrich, St. Louis, MO, USA) with 60% 2-propanol (Sigma-Aldrich) for 20 min at 37 °C, and then rinsed with distilled water. The sections were mounted with glycerinated gelatin and photographed with a microscope (Nikon, Japan). The integral optical density (IOD) was quantified by the Image Pro Plus software (Media Cybernetics, Bethesda, MD, USA).

### 4.4. OA/BSA Complex Solution Preparation

OA/BSA complex solution was prepared according to a previously described method [43]. 100 mM OA stock solution was prepared in 0.1 N NaOH by heating at 70 °C in a shaking water bath. In a water bath at 55 °C, a 10% (*w*/*v*) FFA-free BSA solution was prepared in ddH_2_O. 20 mM OA containing 10% BSA was diluted in the culture medium to obtain the desired final concentration. The OA/BSA complex solution was sterile-filtered through a 0.45 μm pore membrane filter and stored at −20 °C.

### 4.5. Cell Culture

HepG2 cells were routinely cultured in DMEM (Gibco, Waltham, MA, USA) supplemented with 10% FBS in an incubator under an atmosphere of 5% CO_2_ at 37 °C. To accumulate fatty acids, HepG2 cells were exposed to OA at a final concentration of 1 mM for 24 h. Then, daidzein or genistein was added to the cultures at the final concentration of 50 μM for another 24 h before Oil Red O staining was performed. To evaluate the suppression function of soy isoflavones, 50 μM genistein was added to the HepG2 cells and incubated for 24 h, and the insulin treatment was 12 h before genistein was added.

### 4.6. Quantitative Realtime PCR

Total RNA was extracted from the tissues using Trizol reagent (Invitrogen, Waltham, MA USA). RNA was subjected to reverse transcription with reverse transcriptase according to the manufacturer’s instructions (Fermentas, Waltham, MA, USA). Quantitative real-time PCR was performed using the Bio-Rad iQ5 system, and the relative gene expression was normalized to internal control as β-Actin. Primer sequences for SYBR Green probes of the target genes are described in Table 4.

### 4.7. Cell Viability Assay

HepG2 cells were plated in 96-well plates. 48 h after daidzein and genistein treatment, cell viability was measured by Cell Counting Kit-8 (CCK-8) system (Dojindo, CK04-11, Minato-ku, Tokyo, Japan) according to the manufacturer’s instructions. Briefly, CCK-8 solution (10 μL per 100 μL of medium in each well) was added, and the plates were then incubated at 37 °C for 1 h. The absorbance of each well was read at 450 nm using a microplate reader (Thermo, Waltham, MA, USA).

### 4.8. Western Blotting

Standard Western blotting procedures were carried out with the following antibodies: anti-ATP citrate lyase antibody (abcam, ab157098, Cambridge, MA, USA), anti-adipose triglyceride lipase antibody (abcam, ab109251), anti-hormone sensitive lipase antibody (Santa Cruz Biotechnology, sc-25843, Dallas, Texas, USA), anti-ACSL1 antibody (abcam, ab177958), anti-Phospho-Akt (Ser473) (CST, mAb #4060), anti-Phospho-Akt (Thr308) (CST, mAb #13038, Danvers, MA, USA), anti-Akt (CST, mAb #4691), anti-phospho-ribosomal protein S6 (Ser240/Ser244) (Millipore, 07-2113, Billerica, MA, USA), and anti-β-actin (Santa Cruz Biotechnology, sc-47778).

### 4.9. Statistical Analysis

Data represent the mean and standard error of the mean (SEM). One-way ANOVA and *post hoc* tests were performed for all statistical significance analysis using GraphPad Prism software (GraphPad Software, Inc., La Jolla, CA, USA). * *p* < 0.05, ** *p* < 0.01.

## 5. Conclusions

In conclusion, we have shown that the consumption of soy isoflavones could suppress the activity of mTORC1 via the AKT pathway, resulting in a decreased lipogenesis and adipogenesis, and an enhanced lipolysis and β-oxidation in DIO male rats. We reveal a new perspective of soy isoflavones in controlling lipid metabolism of obesity, and this will be important for clinical treatment of lipid abnormalities related disorders.

## Figures and Tables

**Table 1:** Composition of the basal diets.

Ingredients	Content
Corn	54.0%
Fish meal	6.0%
Wheat bran	14.0%
Alfalfa meal	13.0%
Cotton meal	10.0%
Limestone	1.00%
Dicalcium phosphate	0.2%
Dodium chloride	0.3%
Vitamin & mineral	1.5%

**Table 2:** Composition of the experimental diets from week eight to week 12.

Groups	Control Groups (Ctr, *n* = 10)	Obesity Groups (OB, *n* = 15)	Low-Dose Soy Isoflavones (LSI, *n* = 9)	Middle-Dose Soy Isoflavones (MSI, *n* = 15)	High-Dose Soy Isoflavones (HIS, *n* = 15)
Diets	Basal diets	Basal diets	Basal diets + 50 mg/kg soy isoflavones	Basal diets + 150 mg/kg soy isoflavones	Basal diets + 400 mg/kg soy isoflavones

**Table 3:** Composition of the soy isoflavone extracts.

Compounds	Content
Daidzin	50.98%
Glycitin	30.36%
Genistein	8.80%
Daidzein	1.24%
Genistin	0.06%
Total isoflavones	91.64%

**Table 4:** Primers used for the real-time PCR analysis. The amplification efficiency and specificity were confirmed before real-time PCR assay.

Gene	Primers
*SREBP-1c*	Fr 5’-TGGACTACTAGTGTTGGCCTGCTT-3’
	Rv 5’-ATCCAGGTCAGCTTGTTTGCGATG-3’
*ACC-1*	Fr 5’-ATTGTGGCTCAAACTGCAGGT-3’
	Rv 5’-GCCAATCCACTCGAAGACCA-3’
*ACC-2*	Fr 5’-CAACATCCGTCAGACGACCTC-3’
	Rv 5’-CGGACTCGTTGGTGATGAAGA-3’
*FASN*	Fr 5’-TCCCAGGTCTTGCCGTGC-3’
	Rv 5’-GCGGATGCCTAGGATGTGTGC-3’
*ACSL1*	Fr 5’-GGTGCTTCAGCCTACCATCTTCC-3’
	Rv 5’-AATCCAACAGCCATCGCTTCACT-3’
*ACSL4*	Fr 5’-TATGGGCTGACAGAATCATG-3’
	Rv 5’-CAACTCTTCCAGTAGTGTAG-3’
*PPARα*	Fr 5’-ACTCGCAGGAAAGACTAGCA-3’
	Rv 5’-AGCAGTGGAAGAATCGGACC-3’
*HSL*	Fr 5’-TCAGGTGTCTTTGCGGGTAT-3’
	Rv 5’-CTTGTGCGGAAGAAGATGCT-3’
*ATGL*	Fr 5’-TCACCAACACCAGCATCCA-3’
	Rv 5’-GCACATCTCTCGAAGCACCA-3’
*PPARγ*	Fr 5’-CATTCGCATCTTTCAGGG-3’
	Rv 5’-GGACGCCATACTTTAGGA-3’
*ACL*	Fr 5’-GCAGCACGTGATCCATGAAT-3’
	Rv 5’-GTGGGATGCTGGACAACATC-3’
*β-Actin*	Fr 5’-GTACCACTGGCATTGTGATG-3’
	Rv 5’-ATCTTCATGGTGCTAGGAGC-3’

## Abbreviations

The following abbreviations are used in this manuscript:

DIO	diet induced obesity
mTORC1	mechanistic target of rapamycin complexes 1
Ctr	Control
H and E staining	hematoxylin and eosin staining
OA	oleic acid
OB	obesity control group
LSI	low dose of soy isoflavones
MSI	middle dose of soy isoflavones
HSI	high dose of soy isoflavones
TG	triglycerides
LDL	low-density lipoprotein
SREBP1	Sterol regulatory element-binding transcription factor 1
ACC	Acetyl-CoA carboxylase
ACL	ATP citrate lyase
FASN	Fatty acid synthase
ACSL	Long chain fatty acid-CoA ligase
ATGL	Adipose triglyceride lipase
HSL	Hormone sensitive lipase
PPARs	peroxisome proliferator-activated receptors
